# Characteristics of quantitative dynamic contrast-enhanced magnetic resonance imaging for orbital space-occupying lesions: A retrospective case series study

**DOI:** 10.1371/journal.pone.0332199

**Published:** 2025-09-12

**Authors:** Liang Zhao, Zhenfeng Guo, Xiaodong Ji, Fengyuan Sun, Shuang Xia

**Affiliations:** 1 Tianjin Key Laboratory of Retinal Functions and Diseases, Tianjin Branch of National Clinical Research Center for Ocular Disease, Eye Institute and School of Optometry, Tianjin Medical University Eye Hospital, Tianjin, China; 2 Tianjin Beichen Hospital, Tianjin, China; 3 Tianjin First Center Hospital, Tianjin, China; Al-Nahrain University, IRAQ

## Abstract

**Objective:**

This study aimed to compare the differences in the quantitative parameters of quantitative dynamic contrast-enhanced magnetic resonance imaging (DCE-MRI) across various types of orbital space-occupying lesions and to explore the diagnostic efficacy of DCE-MRI in the diagnosis of malignant orbital tumors.Meanwhile,to compare the differences in the quantitative parameters of DCE-MRI across vascular malformations, benign tumors, and malignant orbital lesions,and to explore the diagnostic efficacy of DCE-MRI introducing a novel grouping strategy that distinguishes vascular malformations from solid tumors.

**Methods:**

In this retrospective case series study, patients were classified into three groups: vascular malformations, benign lesions, and malignant lesions. We analyzed the differences in the quantitative parameters and time-intensity curve (TIC) profiles among the three groups. The diagnostic efficacy of the quantitative parameters in the diagnosis of orbital malignant lesions was analyzed using receiver operating characteristic (ROC) curves.

**Results:**

The differences in TIC compositions among the three groups were statistically significant (*P* < 0.05).The differences in volume transfer constant(Ktrans) value,rate constant(Kep) value,and area under curve(iAUC) among the three groups exhibited statistically significant differences (**P* *< 0.05). All three diagnostic parameters(Ktrans, Kep, and iAUC) demonstrated effectiveness in diagnosing malignant lesions. The area under the ROC curve values for Ktrans, Kep, and iAUC were 0.759, 0.764, and 0.752, respectively, indicating adequate diagnostic value.

**Conclusions:**

The composition ratios of the TIC for vascular malformations, benign lesions, and malignant lesions differed considerably. Ktrans, Kep, and iAUC can serve as valuable references for the differential diagnosis of orbital space-occupying lesions.

## Introduction

Orbital space-occupying lesions can be classified into several categories, including benign and malignant tumors, vascular lesions, cysts, inflammatory lesions, etc. For cysts and inflammation, a clear diagnosis can often be made based on clinical manifestations and imaging findings such as CT and MRI. It is often difficult to assess the benign or malignant nature of tumors.However,making preoperative evaluation of benign and malignant tumors essential for effective treatment. Quantitative dynamic contrast-enhanced magnetic resonance imaging (DCE-MRI) is an examination method that evaluates the physiological microcirculation of tumors. Currently, DCE-MRI is widely utilized in research involving tumors in various anatomical regions, including the breast, pancreas, bone, bladder, prostate, head, neck, liver,and others, as well as in the evaluation of benign and malignant tumors prior to surgery, early screening of tumors and postoperative follow-up [1–6]. This method has yielded promising results and serves as an valuable guide for clinical treatment and evaluation [7–9].^.^ Theoretically, there are differences in vascular density and maturity among orbital vascular malformations, benign tumors and malignant tumors. Therefore, there are also differences in their hemodynamics and related parameters of DCE-MRI [10]. Previous studies have mostly focused on comparing the differences in DCE-MRI-related parameters of benign and malignant tumors. However, a large proportion of orbital space-occupying lesions belong to vascular lesions, especially non-dilated vascular malformations, and their MRI quantitative parameters are different from those of solid tumors. In this study, orbital vascular malformations are grouped separately. To compare the differences in quantitative parameters of DCE-MRI for different types of orbital space-occupying lesions, based on our hypothesis of inflammation, so as to more accurately reflect the differences in relevant parameters of DCE-MRI for different types of orbital space-occupying lesions, explore the diagnostic value of DCE-MRI for orbital space-occupying lesions, and guide clinical diagnosis and treatment as well as prognosis evaluation. To our knowledge, this is one of the first studies to systematically evaluate DCE-MRI pharmacokinetic parameters and time-intensity curve profiles across vascular malformations, benign tumors, and malignant tumors of the orbit, offering a novel approach to differential diagnosis in orbital imaging.

## Methods and materials

### Materials

This study is a retrospective case series conducted in accordance with the principles of the Declaration of Helsinki and has been approved by the Medical Ethics Committee of Tianjin Medical University Eye Hospital (Approval No: 2023KY(L)-02). The data collection period is as follows(2023.5.1–2023.6.30). The study included 86 patients with orbital space-occupying lesions who underwent surgery at our hospital between May 15, 2012, and October 22, 2016. Inclusion criteria mandated that patients received an accurate pathological diagnosis following surgical treatment, underwent DCE-MRI examination prior to surgery, and had complete clinical and imaging data. Written informed consent was obtained from all participants, ensuring ethical compliance throughout the research process. Ethics statement and written informed consent have been uploaded.

### Instruments, equipment, and scanning methods

All examinations were performed using a Magnetom Trio Tim 3.0T superconducting magnetic resonance scanner (Siemens, Germany) with a standard 8-channel head coil. The routine MRI scanning included T1-weighted image (T1WI) transverse axial, coronal, and sagittal scanning, T2WI transverse axial, coronal, and sagittal scanning, and T2 fat suppression sequence scanning. Subsequently, DCE-MRI scanning was carried out.

#### Scanning parameter setting and MRI imaging protocol.

The T1WI, T2WI, and fat suppression images were acquired using fast spin echo (FSE) sequencing. The T1WI horizontal axis parameters were set as follows: time of echo (TE): 8.2 ms; time of repetition (TR): 500 ms; flip angle (FLIP): 180°; field of view (FOV): 180 mm × 180 mm; acquisition matrix: 260 × 213; layer thickness: 2.5 mm; layer spacing: 0 mm. The T1WI coronal scan parameters were: TE: 8.2 ms; TR: 600 ms; FLIP: 70°; FOV: 180 mm × 180 mm; acquisition matrix: 260 × 213; layer thickness: 3.0 mm; layer spacing: 0 mm. The T2WI imaging transverse axial scanning parameters were as follows: TE: 94 ms; TR: 6000 ms; FLIP: 120°; FOV: 180 mm × 180 mm; acquisition matrix: 320 × 288; layer thickness: 2.5 mm; layer spacing: 0 mm. The T2WI coronal scan parameters were set as follows: TE: 84 ms; TR: 4000 ms; FLIP: 120°; FOV: 180 mm × 180 mm; acquisition matrix: 384 × 346; layer thickness: 3.0 mm; layer spacing: 0 mm. The T2WI transverse and axial scan parameters of the fat suppression sequence were: TE: 84 ms; TR: 4000 ms; FLIP: 120°; FOV: 180 mm × 180 mm; acquisition matrix: 320 × 288; layer thickness: 2.5 mm; layer spacing: 0.5 mm.

**Fig 1:**
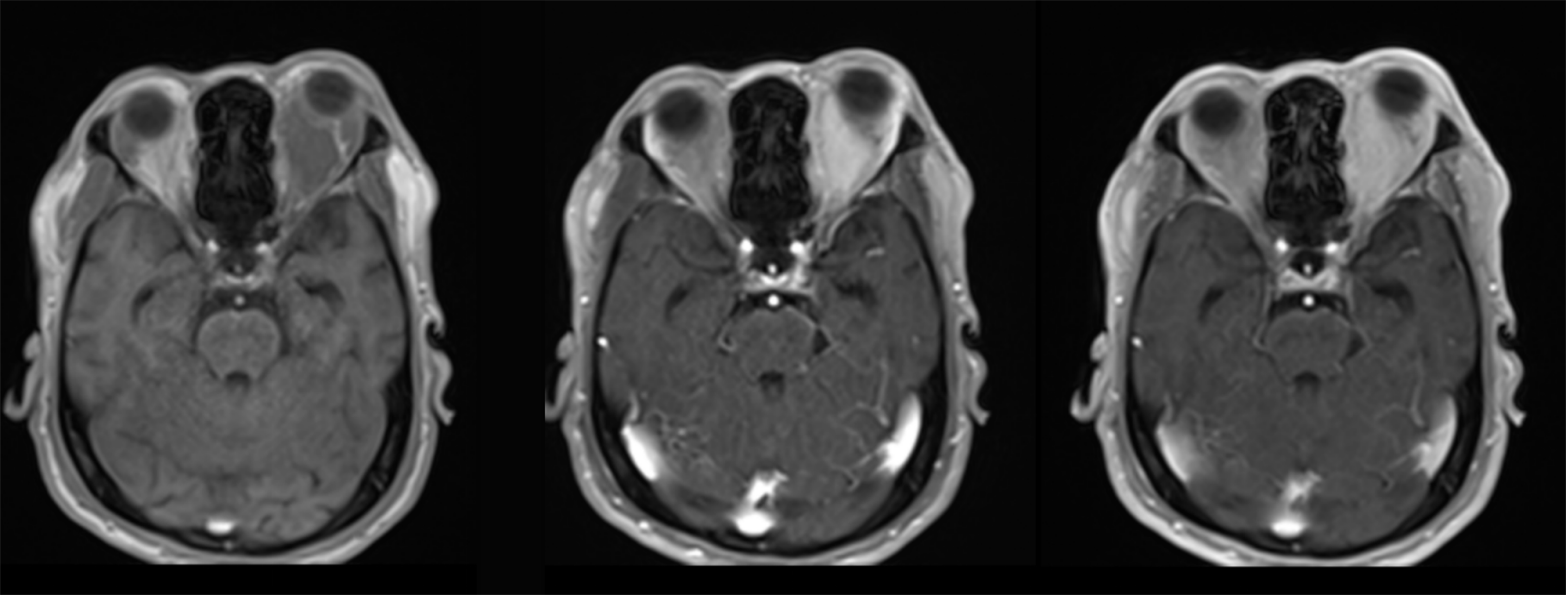
The images of malignant tumors before contrast agent injection, the highest contrast and the contract decreases.

For DCE-MRI, a T1-weighted 3D fast disturbed phase gradient echo (3D-TWIST) sequence with fat suppression sequence was used in the coronal plane. The scanning parameters were as follows: TE: 2.0 ms; TR: 5.0 ms; flip angle: 12°; excitation time: 1; FOV: 170 mm × 240 mm; matrix: 320 × 189; receiving bandwidth: 250 Hz; layer thickness: 3 mm; layer spacing: 0 mm. The scanning range encompassed the orbit of both eyes, extending from the eyelid to the orbital tip. Prior to dynamic contrast-enhanced scanning, the first four frames were acquired to establish a baseline. A T1-weighted reference image was then obtained, after which contrast agent injection commenced from Frame 5. The dose of the Gd-DTPA contrast agent was 0.1 mmol/kg, administered via a high-pressure syringe through the patient’s forearm vein at a rate of 2.0–2.5 ml/s. The contrast agent was rapidly injected over a period of 12 seconds. Following the injection, 20 ml of normal saline was immediately used to flush the intravenous line.A total of 50 scanning frames were acquired with each frame taking 6.4 seconds, resulting in an overall scanning duration of approximately 5 minutes and 23 seconds. So we can obtain MRI images before contrast agent injection, space-occupying enhancement and the gradual weakening of enhancement,as shown in Fig 1.

#### Image post-processing and acquisition.

After the DCE-MRI scanning was completed, the images were transferred to a Siemens 3.0T MR Syngo post-processing workstation. Two experienced radiologists utilized image processing software to analyze and measure the data.

**Fig 2:**
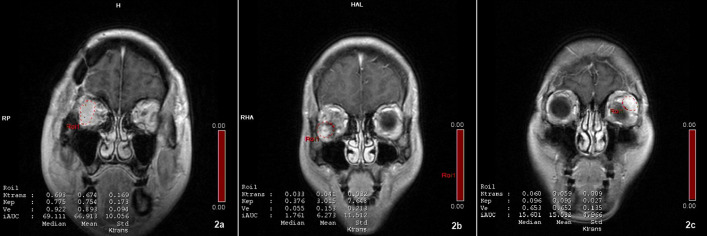
Schematic diagram of the ROI curve:We selected the areas with uniform enhancement as the ROI. a. Lacrimal adenoid cystic carcinoma;b. Cavernous hemangioma;c. Lacrimal pleomorphic adenomas. The data of Ktrans, Kep, Ve and iAUC obtained through measurement are shown in the lower left corner of the figure, including median, mean and standard deviation. The data we collected is the mean.

Selection and drawing of the region of interest (ROI):Manual ROI selection was performed to ensure the accuracy of the measurement data. The selection process involved choosing a tumor area as large as possible while avoiding necrosis, cystic change, hemorrhage, calcification, and surrounding blood vessels to minimize any potential errors. The edge area of the tumor was also avoided to prevent the influence of the partial volume effect, as shown in Fig 2a-c. Following ROI selection, the Syngo workstation automatically generated time-intensity curves (TIC) based on the ROI and calculated the quantitative parameter values. This approach allowed for precise and reliable measurements of the tumor’s characteristics.

**Fig 3:**
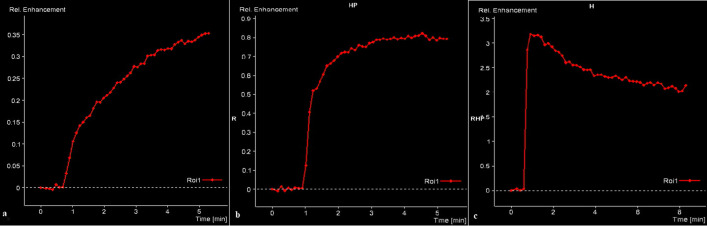
Classification of the TIC curve:a:Type I – Continuous ascent pattern,b:Type II – Platform pattern,c:Type III – Outflow pattern.

TIC classification: The TIC classification in this study was based on the enhancement rate of the lesion at different times after dynamic enhancement. We categorized the TIC into three types. Type I is the continuous rising type, where the signal intensity steadily increases during the period of dynamic observation. Type II is the plateau type, where the curve exhibits a significant increase in signal strength in the early stages of enhancement and remains at a high level for a certain time after the signal strength reaches a peak, displaying a plateau. Type III is the outflow type, where the early signal is significantly enhanced, and after the enhancement reaches the peak, the signal decreases. These three types are illustrated in Fig 3a-c. This TIC classification system allowed for a comprehensive analysis of the tumor’s enhancement pattern and provided valuable information for diagnosis and treatment planning.

Measurement of quantitative parameters: The quantitative parameters that were calculated in this study included the volume transfer constant (Ktrans; min-1), which represents the rate of contrast agent transfer between the plasma and the extravascular extracellular space, the diffusion rate constant of the contrast medium between the tissue and the extracellular space (Kep; min-1), which reflects the rate of contrast agent diffusion from the extravascular extracellular space back into the plasma, the volume fraction of the extravascular extracellular space (Ve), which represents the proportion of the tissue volume occupied by the extravascular extracellular space, and the area under the curve of the semi-quantitative parameter TIC (iAUC), which is a measure of the overall contrast agent uptake in the tumor. These quantitative parameters provided valuable information about the tumor’s microenvironment and vascularization, which can be used for diagnosis and treatment planning.

### Grouping method

To classify the 86 cases of orbital space-occupying lesions based on their pathological results, we adopted a grouping method that involved dividing them into three categories: vascular malformations, benign lesions, and malignant lesions.

### Statistical methods

The statistical analysis of the data was performed using SPSS 19.0 software. A two-way random contingency table analysis was used to compare the constituent ratios of the TIC classification in the vascular malformation group, benign lesion group, and malignant lesion group. A P value of less than 0.05 was considered statistically significant. The quantitative parameters Ktrans, Kep, Ve, and iAUC of the three groups were compared using Kuskal- Waills test or one way ANOVA. A P value of less than 0.05 indicated a statistically significant difference.

Receiver operating characteristic (ROC) curves were generated to calculate the maximum Yoden index (YI = sensitivity + specificity – 1) for each subject. The ideal diagnostic threshold for orbital malignant tumors was determined and the diagnostic efficacy of the DCE-MRI quantitative parameters for orbital malignant tumors was analyzed.

#### Exclusion criteria.

Patients who had not undergone surgery, had not obtained pathology, or had unclear pathology were excluded from the study. Additionally, some orbital space-occupying lesions that were clearly diagnosed, such as cysts, abscesses, and hematomas, were not included in the study.

## Results

### General information and pathological composition of patients for each group

The study included 22 patients in the vascular malformation group, comprising 8 males and 14 females with an age range of 22.0–79.0 years and an average age of 46.5 ± 14.17 years (± s). Seven cases were in the right orbit, and 15 were in the left orbit. Table 1 presents the pathological types of the vascular malformations.

**Table 1:** Pathological types of the vascular malformations.

Pathological types	Number
Cavernous hemangioma	14
Venous hemangioma	8
Total	22

The benign lesion group consisted of 36 patients, including 13 males and 23 females with an age range of 13.0–76.0 years and an average age of 45.7 ± 16.09 years (± s). There were 20 cases in the right orbit, 12 cases in the left orbit, and 4 cases in both orbits. Table 2 presents the various pathological types of the benign lesions.

**Table 2:** Pathological types of the benign lesions.

Pathological types	Number
Orbital nonspecific inflammation	12
Schwannomas	6
Meningioma	5
Solitary fibromas	4
Lacrimal pleomorphic adenomas	4
Inverted papilloma	1
Pigmented neurofibromas	1
Ductal adenoma	1
Eosinophilic granulomas	1
Lipoma	1
Total	36

The malignant lesion group consisted of 28 patients, including 15 males and 13 females with an age range of 26.0–85.0 years and an average age of 55.7 ± 15.52 years (± s). There were 12 cases in the right orbit, 15 cases in the left orbit, and one case in both orbits. The pathological types of the malignant lesions are listed in Table 3.

**Table 3:** Pathological types of the malignant lesions.

Pathological types	Number
Lacrimal adenoid cystic carcinoma	10
Lymphoma	9
Multiple myeloma	1
Basal cell carcinoma	1
Squamous cell carcinoma	1
Meningiomas (malignant)	1
Mucinous adenocarcinoma	1
Fibrosarcoma	1
Fibromyxoid fibrosarcoma	1
Moderately low grade adenocarcinoma	1
Centrally differentiated tubular adenocarcinoma	1
Total	28

### Statistical results of TIC type composition ratio for each group

**Fig 4:**
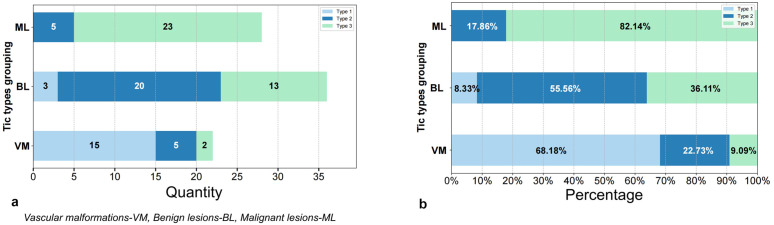
a-b.a: the bar chart;b: the percentage stacking bar chart.

TheTICs of the vascular malformation group were mostly type I, while those of the benign lesion group mainly consisted of type II and type III. The malignant lesion group primarily contained type III TICs. The difference was found to be statistically significant, with a χ2 value of 55.520 and P value of 0.000 (P < 0.05), as shown in Table 4 and Fig 4a-b.

**Table 4:** Comparison of the composition ratios of the three groups of TIC curves.

TIC types Grouping	Type I	Type II	Type III	χ2 value
Vascular malformations	15	5	2	55.520^*^
Benign lesions	3	20	13	
Malignant lesions	0	5	23	

P-values were derived from Chi-Square tests.

* Statistical significance(P < 0.05).

### Comparative statistical results of quantitative parameters for each group

The average Ktrans value in the vascular malformation group was significantly lower than that in the benign lesion group, while the average Ktrans value in the benign lesion group was also lower than that in the malignant lesion group. The differences in Ktrans values among the three groups were statistically significant (P < 0.05). Additionally, the Kep values for both the vascular malformation group and the benign lesion group were lower than those of the malignant lesion group, with this difference also being statistically significant (P < 0.05). However, there was no statistically significant difference in Kep values between the vascular malformation group and the benign lesion group. When comparing Ve values among the three groups, no statistically significant differences were observed (P > 0.05). The iAUC values in the vascular malformation group were lower than those in both the benign and malignant lesion groups, with this difference being statistically significant (P < 0.05). There was no statistically significant difference in iAUC values between the benign and malignant lesion groups.

Table 5 shows the comparative statistical results of the DCE-MRI quantitative parameters among vascular malformations, benign lesions and malignant lesions.

**Table 5:** Comparison of DCE-MRI quantitative parameters among three groups.

	Vascular malformations	Benign lesions	Malignant lesions	H/F
K-trans	0.168 (0.160)^a^	0.227 (0.211)^b^	0.312 (0.157)^c^	22.718^#*^
Kep	0.287 (0.223)^a^	0.391 (0.324)^a^	0.559 (0.276)^b^	20.697^#*^
ve	0.631 ± 0.161	0.646 ± 0.145	0.596 ± 0.154	0.893^△^
iAUC	9.234 (10.740)^a^	28.819(22.855)^b^	35.884 (10.986)^b^	36.215^#*^

Note: ^#^ Kuskal-Waills test, ^△^one way ANOVA, comparison between groups Bonferoni.

a.b.c The same letter was not statistically significant between groups.

* Statistical significance(P < 0.05).

### Evaluation of the DCE-MRI quantitative parameters in the diagnosis of orbital malignant tumors

**Fig 5:**
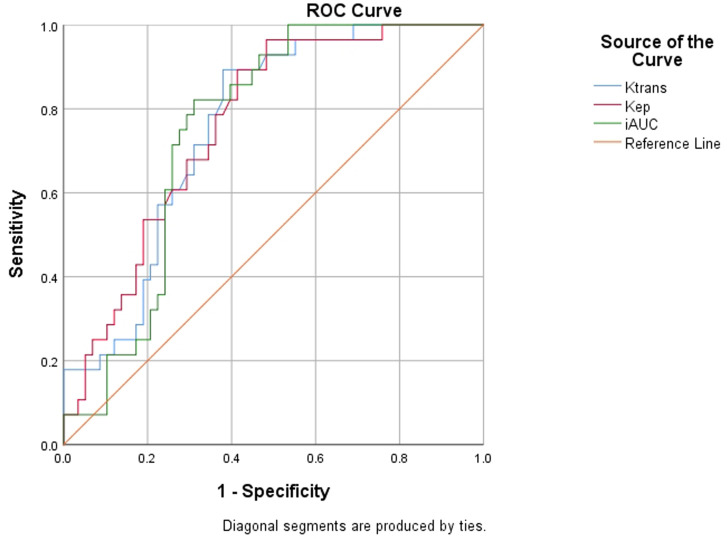
ROC curves of Ktrans values, Kep values and iAUC:The curves showed that the three diagnostic methods were all effective, with medium diagnostic values.

ROC curves for Ktrans, Kep, and iAUC were generated based on the gold standard of benign and malignant pathological results, as shown in Fig 5. The diagnostic validity test for all three parameters yielded a P value of less than 0.05, indicating that the three diagnostic methods were all effective with medium diagnostic values, as presented in Table 6.

**Table 6:** Diagnostic efficacy of DCE-MRI quantitative parameters for orbital malignant lesions.

Quantitative parameters	Area under the curve	The most Yoden Index	Diagnostic cut-offs	Sensitivity(%)	Specificity(%)	P
Ktrans	0.759	0.514	0.234	89.3	62.1	*
Kep	0.764	0.418	0.317	96.4	51.7	*
iAUC	0.752	0.511	29.223	82.1	69.0	*

P-values were derived from receiver operating characteristic (ROC) curves.

* Statistical significance(P < 0.05).

## Discussion

Quantitative DCE-MRI involves obtaining a series of images through continuous scanning and acquisition of spoiled GRE T1WI sequences following the injection of a contrast agent via a high-pressure injector. A two-compartment pharmacokinetic model is then employed to calculate quantitative parameters related to vascular physiology, allowing for dynamic observation of the distribution and excretion process of the contrast agent in the tumor. This method directly reflects the physiological characteristics of microvessels in the affected tissue [11,12].

This study compared the quantitative parameters of DCE-MRI across different types of orbital space-occupying lesions. The results indicated that the Ktrans, and iAUC values of vascular malformations were lower than those of benign and malignant lesions, while the differences in the Ve value were not statistically significant.Notably, there was no significant difference in Kep between the vascular malformation and benign lesion groups. Furthermore,Ktrans and Kep values in benign lesions were lower than those in malignant lesions, aligning with previous research on head and neck and chest tumors [13–15]. Ktrans and Kep are indicators that reflect tissue blood flow, capillary permeability, and capillary surface area. Increased blood flow and larger vascular surface area lead to stronger permeability, resulting in higher Ktrans and Kep values, and vice versa. Malignant tumor cells exhibit vigorous proliferation, leading to the formation of numerous new blood vessels, increased microvessel density, and greater blood flow, resulting in higher Ktrans and Kep values than benign tumors. In contrast, vascular malformations, characterized by highest maturity, result in lower Ktrans and Kep values than tumor lesions [16–18]. Our study further demonstrated that Ktrans values significantly differed among the three groups, suggesting that Ktrans is particularly valuable for the differential diagnosis of these lesions. Since orbital vascular malformations are generally easier to diagnose than solid tumors, we grouped vascular malformations separately and compared the DCE-MRI quantitative parameters of benign and malignant solid tumors, providing more clinical reference value. The findings confirmed notable differences in the quantitative parameters of DCE-MRI among the three groups; however, the limited number of cases included in this study highlights the need for more extensive research in the future. Ve represents the ratio of extracellular blood vessel volume within a unit volume of tissue. Despite the high density and enlarged nuclei of malignant tumor cells, the Ve value did not significantly differ from that of benign tumors, which may be attributed to the small sample size or tissue edema around the lesion [19].Therefore, Ve appears to have limited significance in the differentiation of benign and malignant diseases, necessitating further investigation into its underlying causes and physiological relevance [20]. The iAUC, being a semi-quantitative parameter, has certain limitations, and the results in existing literature vary considerably.Consequently, it is suggested that the clinical significance of quantitative parameters is superior to that of semi-quantitative parameters [14].

This study compared the types of TIC across the three groups. The TICs in the vascular malformation group were predominantly exhibited type I, while the benign lesion group primarily consisted of type II and III.In contrast, the malignant lesion group typically displayed type III TICs.The vascular malformation group included cavernous hemangioma and venous hemangioma, which are characterized as non-dilated venous malformations with relatively mature blood vessels, slow blood flow rate, and gradual enhancement curve rises. Due to the small cellular components of these tumors and the larger extracellular space within the blood vessel compared to the tumor itself, the contrast agent accumulates in the extracellular space over an extended period.Consequently, the reflux is slow, resulting in predominantly type I TICs.On the other hand, malignant tumors exhibit high neovascularization density, low vascular maturity, increased permeability, and rapid tissue enhancement. The high density of malignant tumor cells and the limited extracellular space surrounding the blood vessels often lead to rapid contrast agent reflux, resulting in predominantly type III TICs. These findings are consistent with previous research [21–23].

Although this study yielded positive results, some tumor measurement data still exhibited irregularities. In the vascular malformation group, two cases of venous hemangioma demonstrated an outflow-type TIC,while four cases of cavernous hemangioma displayed a plateau-type TIC.This suggests the presence of nutrient vessels and immature vascular tissue with a rapid flow rate in certain venous hemangiomas and cavernous hemangiomas. However, further histopathological studies are necessary to confirm these findings. In the benign lesion group, a total of 13 tumors exhibited an outflow-type TIC with high Ktrans, Kep, and iAUC values. This group included four meningiomas, five cases of orbital nonspecific inflammatory reactions (including two IgG4-related ophthalmopathies), three solitary fibrous tumors, and one inverted papilloma. Although meningiomas are classified as benign tumors, they possess high cell density and exhibit rich, rapid blood flow, leading to DCE-MRI quantitative parameters and TICs that often resemble those of malignant tumors, characterized by high Ktrans and Kep values alongside outflow-type TICs.Similar findings have been reported in other studies [24,25]. Orbital meningiomas often communicate with the brain and generally occur at the sphenoid spine, facilitating clinical diagnosis. Solitary fibrous tumors are characterized by areas rich in both cells and fibers featuring abundant blood vessels, thin-walled tube, and expanded, convoluted lumens that resemble a staghorn shape. Local necrosis may also occur in some solitary fibrous tumors, resulting in areas of uneven and pronounced enhancements on enhanced MRI, which generally correspond to cell-concentrated and vascular enrichment areas. IgG4-associated ophthalmopathy is marked by significant infiltration of lymphoplasmacytic cells, similar to lymphoma, resulting in higher cell density, reduced extracellular space, and earlier contrast agent reflux.These factors influence its Kep value and TIC type [8,25]. Some studies investigating the diffusion-weighted imaging (DWI) characteristics of orbital tumors indicate that the apparent diffusion coefficient (ADC) value of IgG4-associated ophthalmopathy is lower than benign lymphoproliferative lesions and similar to malignant lymphoma [26]. Studies on the pancreas and urethra also suggest that the diffusion of water molecules in IgG4-related diseases is limited, confirming the hypothesis that the density of IgG4-related ophthalmopathy cells is high and the extracellular space is small, affecting the Kep value and TIC [23].

The ROC curve serves as a graphical representation of the diagnostic efficacy for each threshold value. The point nearest to the the upper left corner of the ROC curve represents the threshold where the sum of the false positive rate and the false negative rate is the minimized, thereby yielding the maximum Youden’s index lowest. The value range of the area under the ROC curve is 0.5–1.0. A value between 0.5–0.7 indicates low diagnostic ability, while a value between 0.7–0.9 indicates medium diagnostic value, and values above 0.9 indicate the highest diagnostic value [27–29]. In this study, we plotted the ROC curves for the DCE-MRI quantitative parameters Ktrans, Kep, and iAUC confirming that these parameters effectively diagnose orbital malignant lesions with moderate diagnostic value. However, our findings revealed certain discrepancies in the diagnostic efficacy of the DCE-MRI quantitative parameters compared to existing literature [30]. Moreover, relying solely on DCE-MRI quantitative parameters alone as diagnostic indices for orbital malignant tumors may not achieve both high specificity and high sensitivity.Additionally, variations in MRI instrument parameter settings and errors in manual measurements are also factors that cause inconsistencies in the results of various studies.

## Conclusions

Accurately evaluating the nature of orbital space-occupying lesions is essential for developing effective treatment plans and designing surgical procedures. Imaging examinations play a vital role in determining the nature of lesions before surgery. DCE-MRI provides a new diagnostic basis for the qualitative assessment of orbital space-occupying lesions by directly reflecting the biological characteristics of microvessels in the lesion tissue. However, to enhance the clinical application of DCE-MRI, more thorough research is necessary to refine the classification of orbital masses and establish accurate and standardized diagnostic indicators. This advancement will empower clinicians to make more informed decisions regarding treatment options and ultimately improving patient outcomes.

## Limitations

This study is a single-center study with a small sample size, a wide variety of diseases, and a single ethnic group of the research subjects. These are the limitations of this study. In future research, we will increase the number of research centers, expand the sample size, and conduct in-depth studies on single diseases to conduct more detailed and in-depth research on DCE-MRI of orbital space-occupying lesions

## Supporting information

S1 Table Original data of table2. (XLSX)

S2 Table Original data of table3. (XLSX)

S3 Table Original data of table4. (XLSX)

S4 Table Original data of table5 and table6. (XLSX)

S1 File Minimal Data. (RAR)

## References

[1] Park H, Kim SH, Kim JY. Dynamic contrast-enhanced magnetic resonance imaging for risk stratification in patients with prostate cancer. Quant Imaging Med Surg. 2022;12(1):742–51. doi: 10.21037/qims-21-455 34993115 PMC8666742

[2] Jing H, Yan X, Li J, Qin D, Zhang N, Zhang H. The Value of Dynamic Contrast-Enhanced Magnetic Resonance Imaging (DCE-MRI) in the Differentiation of Pseudoprogression and Recurrence of Intracranial Gliomas. Contrast Media Mol Imaging. 2022;2022:5680522. doi: 10.1155/2022/5680522 35935318 PMC9337951

[3] Su W, Hou X, Yu B. Value of dynamic contrast-enhanced magnetic resonance imaging in combination with mammography for screening early-stage breast cancer. Afr Health Sci. 2023;23(2):290–7. doi: 10.4314/ahs.v23i2.33 38223626 PMC10782360

[4] Zhang Y, He D, Liu J, Wei Y-G, Shi L-L. Preoperative prediction of macrotrabecular-massive hepatocellular carcinoma through dynamic contrast-enhanced magnetic resonance imaging-based radiomics. World J Gastroenterol. 2023;29(13):2001–14. doi: 10.3748/wjg.v29.i13.2001 37155523 PMC10122786

[5] Wang N, Gaddam S, Xie Y, Christodoulou AG, Wu C, Ma S, et al. Multitasking dynamic contrast enhanced magnetic resonance imaging can accurately differentiate chronic pancreatitis from pancreatic ductal adenocarcinoma. Front Oncol. 2023;12:1007134. doi: 10.3389/fonc.2022.1007134 36686811 PMC9853434

[6] Li W-Z, Wu G, Li T-S, Dai G-M, Liao Y-T, Yang Q-Y, et al. Dynamic contrast-enhanced magnetic resonance imaging-based radiomics for the prediction of progression-free survival in advanced nasopharyngeal carcinoma. Front Oncol. 2022;12:955866. doi: 10.3389/fonc.2022.955866 36338711 PMC9627984

[7] Russo C, Strianese D, Perrotta M, Iuliano A, Bernardo R, Romeo V, et al. Multi-parametric magnetic resonance imaging characterization of orbital lesions: a triple blind study. Semin Ophthalmol. 2020;35(2):95–102. doi: 10.1080/08820538.2020.1742358 32298217

[8] Jittapiromsak N, Hou P, Liu H-L, Sun J, Schiffman JS, Chi TL. Dynamic contrast-enhanced MRI of orbital and anterior visual pathway lesions. Magn Reson Imaging. 2018;51:44–50. doi: 10.1016/j.mri.2018.04.016 29709464

[9] Petralia G, Summers PE, Agostini A, Ambrosini R, Cianci R, Cristel G, et al. Dynamic contrast-enhanced MRI in oncology: how we do it. Radiol Med. 2020;125(12):1288–300. doi: 10.1007/s11547-020-01220-z 32415476

[10] Petea-Balea R, Lenghel M, Rotar H, Dinu C, Bran S, Onisor F, et al. Role of dynamic contrast enhanced magnetic resonance imaging in the diagnosis and management of vascular lesions of the head and neck. Bosn J Basic Med Sci. 2022;22(2):156–63. doi: 10.17305/bjbms.2021.6019 34420512 PMC8977080

[11] Bagher-Ebadian H, Brown SL, Ghassemi MM, Nagaraja TN, Valadie OG, Acharya PC, et al. Dynamic contrast enhanced (DCE) MRI estimation of vascular parameters using knowledge-based adaptive models. Sci Rep. 2023;13(1):9672. doi: 10.1038/s41598-023-36483-9 37316579 PMC10267191

[12] Li X, Huang W, Holmes JH. Dynamic Contrast-Enhanced (DCE) MRI. Magn Reson Imaging Clin N Am. 2024;32(1):47–61. doi: 10.1016/j.mric.2023.09.001 38007282

[13] Vaid S, Chandorkar A, Atre A, Shah D, Vaid N. Differentiating recurrent tumours from post-treatment changes in head and neck cancers: does diffusion-weighted MRI solve the eternal dilemma?. Clin Radiol. 2017;72(1):74–83. doi: 10.1016/j.crad.2016.09.019 27789026

[14] O’Shaughnessy E, Cossec CL, Mambour N, Lecoeuvre A, Savatovsky J, Zmuda M, et al. Diagnostic Performance of Dynamic Contrast-Enhanced 3T MR Imaging for Characterization of Orbital Lesions: Validation in a Large Prospective Study. AJNR Am J Neuroradiol. 2024;45(3):342–50. doi: 10.3174/ajnr.A8131 38453407 PMC11286117

[15] Dong H, Kang L, Cheng S, Zhang R. Diagnostic performance of dynamic contrast-enhanced magnetic resonance imaging for breast cancer detection: An update meta-analysis. Thorac Cancer. 2021;12(23):3201–7. doi: 10.1111/1759-7714.14187 34668649 PMC8636198

[16] Ang T, Juniat V, Patel S, Selva D. Evaluation of orbital lesions with DCE-MRI: a literature review. Orbit. 2024;43(3):408–16. doi: 10.1080/01676830.2022.2149819 36437715

[17] Hoffmann C, Mohr C, Johansson P, Eckstein A, Huettmann A, von Tresckow J, et al. MRI-based long-term follow-up of indolent orbital lymphomas after curative radiotherapy: imaging remission criteria and volumetric regression kinetics. Sci Rep. 2023;13(1):4792. doi: 10.1038/s41598-023-31941-w 36959374 PMC10036339

[18] Hou Y, Xie X, Chen J, Lv P, Jiang S, He X, et al. Bag-of-features-based radiomics for differentiation of ocular adnexal lymphoma and idiopathic orbital inflammation from contrast-enhanced MRI. Eur Radiol. 2021;31(1):24–33. doi: 10.1007/s00330-020-07110-2 32789530

[19] Kim S, Loevner L, Quon H, Sherman E, Weinstein G, Kilger A, et al. Diffusion-weighted magnetic resonance imaging for predicting and detecting early response to chemoradiation therapy of squamous cell carcinomas of the head and neck. Clin Cancer Res. 2009;15(3):986–94. doi: 10.1158/1078-0432.CCR-08-1287 19188170 PMC2673914

[20] Guo W, Luo D, Lin M, Wu B, Li L, Zhao Y, et al. Pretreatment Intra-Voxel Incoherent Motion Diffusion-Weighted Imaging (IVIM-DWI) in Predicting Induction Chemotherapy Response in Locally Advanced Hypopharyngeal Carcinoma. Medicine (Baltimore). 2016;95(10):e3039. doi: 10.1097/MD.0000000000003039 26962824 PMC4998905

[21] Sun B, Song L, Wang X, Li J, Xian J, Wang F, et al. Lymphoma and inflammation in the orbit: Diagnostic performance with diffusion-weighted imaging and dynamic contrast-enhanced MRI. J Magn Reson Imaging. 2017;45(5):1438–45. doi: 10.1002/jmri.25480 27649521

[22] Ro S-R, Asbach P, Siebert E, Bertelmann E, Hamm B, Erb-Eigner K. Characterization of orbital masses by multiparametric MRI. Eur J Radiol. 2016;85(2):324–36. doi: 10.1016/j.ejrad.2015.11.041 26781137

[23] Calandriello L, Grimaldi G, Petrone G, Rigante M, Petroni S, Riso M, et al. Cavernous venous malformation (cavernous hemangioma) of the orbit: Current concepts and a review of the literature. Surv Ophthalmol. 2017;62(4):393–403. doi: 10.1016/j.survophthal.2017.01.004 28131871

[24] Kalin-Hajdu E, Colby JB, Idowu O, Grumbine FL, Kang JM, Hirabayashi KS, et al. Diagnosing Distensible Venous Malformations of the Orbit With Diffusion-weighted Magnetic Resonance Imaging. Am J Ophthalmol. 2018;189:146–54. doi: 10.1016/j.ajo.2018.02.005 29458038

[25] Li J, Zhou C, Qu X, Du L, Yuan Q, Han Q, et al. Perilesional dominance: radiomics of multiparametric MRI enhances differentiation of IgG4-Related ophthalmic disease and orbital MALT lymphoma. BMC Med Imaging. 2025;25(1):238. doi: 10.1186/s12880-025-01771-5 40597761 PMC12220517

[26] Connolly M, Srinivasan A. Diffusion-Weighted Imaging in Head and Neck Cancer: Technique, Limitations, and Applications. Magn Reson Imaging Clin N Am. 2018;26(1):121–33. doi: 10.1016/j.mric.2017.08.011 29128000

[27] Tsuchiya M, Masui T, Otsuki Y, Sakahara H. Mesenchymal chondrosarcoma of the orbit: imaging features of CT and MRI. Br J Radiol. 2018;91(1090):20170579. doi: 10.1259/bjr.20170579 29975155 PMC6350477

[28] Paul K, Huelnhagen T, Oberacker E, Wenz D, Kuehne A, Waiczies H, et al. Multiband diffusion-weighted MRI of the eye and orbit free of geometric distortions using a RARE-EPI hybrid. NMR Biomed. 2018;31(3):10.1002/nbm.3872. doi: 10.1002/nbm.3872 29315932

[29] Surov A, Kim JY, Aiello M, Huang W, Yankeelov TE, Wienke A, et al. Associations Between Dynamic Contrast Enhanced Magnetic Resonance Imaging and Clinically Relevant Histopathological Features in Breast Cancer: A Multicenter Analysis. In Vivo. 2022;36(1):398–408. doi: 10.21873/invivo.12717 34972741 PMC8765187

[30] Sun B, Song L, Wang X, Li J, Xian J, Wang F, et al. Lymphoma and inflammation in the orbit: Diagnostic performance with diffusion-weighted imaging and dynamic contrast-enhanced MRI. J Magn Reson Imaging. 2017;45(5):1438–45. doi: 10.1002/jmri.25480 27649521

